# Child Death in a Resource-Limited Setting: A Simulation Case for Pediatric Residents to Prepare for Global Health Electives

**DOI:** 10.15766/mep_2374-8265.11341

**Published:** 2023-09-01

**Authors:** Duncan K. Hau, Joy D. Howell, Adetunbi Ayeni, Michael J. Alfonzo, Kevin Ching

**Affiliations:** 1 Rotation Director, Global Health Elective Pediatric Residency Program, and Assistant Professor of Clinical Pediatrics, Weill Cornell Medical College; 2 Assistant Dean for Diversity and Student Life and Professor of Clinical Pediatrics, Weill Cornell Medical College; 3 Associate Fellowship Program Director, Pediatric Emergency Medicine Fellowship, NewYork-Presbyterian Brooklyn Methodist Hospital; 4 Director, Pediatric Emergency Medicine Global Health Fellowship Track, and Assistant Professor of Clinical Emergency Medicine and Pediatrics, Weill Cornell Medical College; 5 Medical Director, Weill Cornell Medicine NewYork-Presbyterian Simulation Center, and Associate Professor of Emergency Medicine and Pediatrics, Weill Cornell Medical College

**Keywords:** Cultural Awareness, Case-Based Learning, Global Health, Hospital Medicine, Pediatrics, Simulation

## Abstract

**Introduction:**

Pediatric residents are increasingly pursuing global health electives. Differences in cultural norms and management around pediatric deaths in resource-limited settings can be emotionally overwhelming for residents. Educational resources are needed to better equip them for handling these stressful situations. We developed a predeparture simulation child death case to prepare pediatric residents for their global health elective

**Methods:**

The simulation module included a clinical case followed by a multidisciplinary structured debriefing. The case featured a 5-year-old, malnourished child in hypovolemic shock who clinically deteriorates and dies. After obtaining a history and performing a physical examination, residents were expected to diagnose severe malnutrition, treat hypovolemic shock, and decide how far to extend resuscitation with the limited resources. Upon returning from abroad, residents were invited to complete a survey on the utility of the simulation case module in preparing for their elective.

**Results:**

Twenty-nine residents participated in the simulation case module, and 18 completed the survey. Seventeen agreed or strongly agreed that the simulation module was a useful tool for preparation (*Mdn* = 4.5 on a 5-point Likert scale). Residents reflected that the simulation module helped manage expectations and provided them with an understanding of the cross-cultural differences in managing pediatric deaths in a resource-limited setting.

**Discussion:**

Pediatric residents trained in resource-rich countries do not encounter death often. Postgraduate training programs could consider simulations like this one to prepare such residents for cross-cultural differences in managing pediatric deaths and build resiliency to operate in resource-limited settings.

## Educational Objectives

By the end of this activity, learners will be able to:

1. Prepare for cross-cultural differences that may impact the delivery of health care as part of the care team during a global health elective.
2. Demonstrate heightened cross-cultural awareness of different approaches to managing child death in a resource-limited setting.

## Introduction

Interest in global health training during residency has been steadily growing, and global health electives are increasingly being offered during pediatric residency.^1,2^ Studies have shown that global health electives improve physical examination skills, increase knowledge of tropical medicine, encourage the practice of cost-effective medicine, and enhance cultural competency.^3–7^ However, only 66% of pediatric residency programs offer any predeparture preparation.^1^ It is speculated that lack of preparation can leave residents feeling unprepared to deal with the challenges of working in a resource-limited setting.^8^ One major challenge encountered by pediatric residents is witnessing high rates of child mortality. For example, the under-5 mortality rate in sub-Saharan Africa is around 74 deaths per 1,000 live births, compared to just six deaths per 1,000 live births in Northern America.^9^ Time abroad can be emotionally overwhelming for pediatric residents, and resources are needed to better equip them and build their resiliency for handling stressful situations.^10^

Simulation-based medical education has been shown to be a useful tool for global health preparation.^11–13^ Medical simulations can prepare residents to treat conditions and manage situations rarely encountered where their training is based. For pediatric residents based in resource-rich settings who encounter death of children at relatively low rates, it is important to understand cross-cultural differences in the delivery of health care and in managing child death. For example, differences in cultural norms of when to initiate or stop resuscitation on a child can vary significantly between high-resource and low-resource settings; if pediatric residents are unaware of this, they can experience feelings of frustration and futility. In low-resource settings, local health care providers may deliberately perform fewer lifesaving interventions, such as choosing not to resuscitate a dying child because of the limitations and poor outcomes in their setting. For visiting residents trained in high-resource settings, the default reaction is to do whatever necessary to save a child's life. However, if this approach is applied in low-resource settings, it can end up causing more harm and aggravation for all. It is imperative for visiting residents who may be unfamiliar with local cultural norms and languages of the host institution to be aware of these issues and not be perceived as hegemonic when dealing with the management of a dying child. The literature on simulation of death in preparation for a global health elective is limited.^11^ A search of *MedEdPORTAL* found one publication focused on using simulation to prepare for ethical dilemmas encountered on a global health elective but no cases around managing death.^14^

Conscious of the cultural norms and differences in managing pediatric death in resource-limited settings, we developed a predeparture global health simulation case module to better equip and heighten cross-cultural awareness of pediatric residents going on a global health elective.

## Methods

### Development

Pediatric residents in their third year of training at the NewYork-Presbyterian/Weill Cornell Medical Center (WCMC) residency program in New York City, New York, could participate in a 4-week global health elective based at the Bugando Medical Center (BMC) in Mwanza, Tanzania.^4^ BMC is a public academic tertiary hospital with 1,000 inpatient beds and approximately 3,500 pediatric hospitalizations per year. The under-5 mortality rate in Tanzania is 50 per 1,000 live births, compared to six per 1,000 live births in the US.^9^ Residents rotated through various pediatric units of the BMC, including the pediatric intensive care unit, the neonatal intensive care unit, the general inpatient floor, and a designated regional malnutrition unit.

Prior to departure and 1–2 months before their elective, residents were required to take part in a 1-hour global health simulation case module at the Weill Cornell Medicine NewYork-Presbyterian Simulation Center. Residents were assigned into groups of three to four people per session to work as a team. These sessions were held after work hours for the residents, so as to not conflict with their clinical responsibilities. At our institution, with an average of eight to 12 residents participating in the elective each year, we typically conducted three to four sessions each academic year. Residents had access to a pediatric handbook on caring for children in limited-resource settings during the simulation and to an electronic version of the handbook during their elective.^15^ They were also given an article prior to departure discussing perceptions held by health care professionals in resource-limited settings towards visiting learners.^16^ Salient points from the article were discussed during a debriefing session.

The global health simulation case module was developed by WCMC pediatric faculty who had visited or worked in Tanzania or another sub-Saharan African country and by Tanzanian pediatricians from BMC. The faculty who facilitated the debriefing had experience in global health and medical education. Prior to the simulation, all residents in their second year took part in a 1-day bereavement training session that discussed death in a formal way.

### Equipment

The room, medical equipment, and props were set up to simulate a low-resource hospital setting. We recommend the following equipment for successful implementation:

• Primary: pediatric manikin simulator with brown skin tone.
  ○ Simulated liquid diarrhea (egress from patient's shorts via hidden tubing and large-volume syringe)
• Secondary: additional pediatric manikins with brown skin tone to create a crowded hospital (optional)
• Thermometer
• Pediatric blood pressure cuff (placed in an inconspicuous location)
• Portable pulse oximeter
• Oxygen tank (empty) and nasal cannula
• Empty glucometer box
• ReSoMal (Rehydration Solution for Malnutrition) packets
• Supplies for IV line placement
• Nasal gastric tube
• Syringes and needles of various sizes
• Fluid bags labeled Ringer's lactate with 5% glucose and 10% glucose
• Medications labeled paracetamol, amoxicillin, ceftriaxone, and adrenaline (two doses only)
• MUAC (mid-upper arm circumference) tape
• Clipboard with initial vital signs, anthropometric measurements, and a table for weight-for-height/length for 2- to 5-year-old boys
• Malnutrition Ward room sign

### Personnel

In each simulation, there were three to four pediatric residents, one or more facilitators, two trained standardized patient (SP) actors, and a simulation technician. One SP acted as the child's caretaker and provided pertinent medical history when requested by the residents. The other SP acted as an experienced Tanzanian pediatric nurse and assisted the residents by providing the limited resources available in the hospital. A simulation technician was responsible for changing physical signs on the manikin simulator.

### Implementation

The residents were introduced to the Malnutrition Ward at a resource-limited hospital by a facilitator and told they needed to evaluate a 5-year-old, HIV-infected child brought into the hospital from the local orphanage. The facilitator then left and went behind side curtains in the room to join the simulation technician. Both the facilitator and technician were able to observe and hear the residents. The residents examined the child and interviewed the caretaker to collect information about the child's medical history while communicating with the nurse to request diagnostic tests, equipment, and treatment options. As scripted but undisclosed, the nurse deliberately appeared unhurried when assisting with any requests to procure medical supplies and would leave the patient's room for long periods of time to demonstrate her competing demands of caring for other critically ill children in the hospital.

Initial vital signs and presentation of the child manikin were consistent with compensated hypovolemic shock. Over the next 20 minutes, the child progressively deteriorated despite all forms of medication and fluid resuscitation provided and eventually developed cardiac arrest. The case ended at this point. The full simulation case is presented in Appendix A. Simulation images are provided in Appendix B. A critical actions checklist is presented in Appendix C. Directly following the case, the residents participated in a 40-minute debriefing session.

### Debriefing

Using the PEARLS (Promoting Excellence and Reflective Learning in Simulation) framework, a combination of plus-delta and focused-facilitation methods was utilized to address both the medical management of the child and the emotional reactions of the residents to the child's death.^17^ The plus-delta approach was used to promote the residents’ capacity for self-assessment and to manage perception mismatches, which could be helpful in developing cultural awareness and sensitivity. During the debriefing session, time was spent reflecting on the nurse's unhurried demeanor while the child was decompensating and how this made the residents feel. Focused facilitation was used to probe deeper and explore specific issues around the management of resuscitating a child in a limited-resource setting. Debriefing questions and materials are presented in Appendix D.

### Assessment

As noted, the facilitators completed a critical actions checklist (Appendix C) during the simulation and shared their observations and feedback during the debriefing session. Upon returning to the US from their global health elective, residents were asked to complete a survey assessing their predeparture global health simulation case module (Appendix E). The survey instrument consisted of multiple-choice questions, Likert-scale questions, and an open-ended response question. The assessment was approved by the Weill Cornell Medical College Institutional Review Board (reference number: 1603017051).

## Results

From July 2016 to March 2020, 29 WCMC pediatric residents participated in the predeparture global health simulation case module and completed the global health elective. A survey was sent to all 29 WCMC residents following the elective, and 18 residents completed it. During their 4-week global health elective, four of the 18 residents reported witnessing zero deaths, 12 reported witnessing one to five deaths, and two reported witnessing six to 10 deaths. Eleven of the 18 residents reported participating in a resuscitation event while in Tanzania.

Seventeen of the 18 residents agreed or strongly agreed that the predeparture global health simulation case module was a useful tool for global health training (*Mdn* = 4.5 on a 5-point Likert scale [1 = *strongly disagree,* 5 = *strongly agree*]; Table). Fifteen agreed or strongly agreed that the global health simulation case module was beneficial to their preparation for the emotional aspects encountered during the elective (*Mdn* = 4.0). Sixteen agreed or strongly agreed that the global health simulation case module was beneficial to their preparation for the mortality encountered (*Mdn* = 4.0).

**Table.**
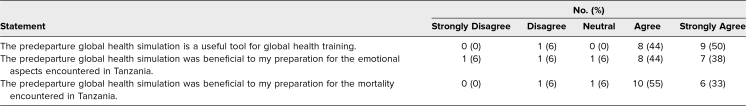
Postelective Resident Evaluation of the Simulation's Usefulness in Preparing for the Global Health Elective (*N* = 18)

Below are selected residents’ experiences from the survey regarding the use of simulated child death for preparation of a global health elective:

• “The pre-departure simulation was probably the single most valuable experience that prepared me for the realities on the ground in Tanzania. Without the sim, I would have had a much harder time adjusting.”
• “I believed the simulation helped manage expectations during real world encounters. However, real world encounters were still emotionally difficult.”
• “It is impossible to simulate all scenarios or fully prepare for the Tanzania experience through sim, but it was an eye-opening experience that allowed us to have a small glimpse before actually getting to Tanzania—very worthwhile.”
• “I thought the simulation was realistic and very helpful to manage my expectations before my Tanzania rotation.”
• “Death was far more prevalent in Tanzania than I've ever experienced here, and the experience of simulating death and how accustomed to it people are there, was helpful.”
• “I think the simulation significantly helped my ability to frame the experience I was about to have in Tanzania, and to re-adjust my expectations. It was tremendously helpful and made me less anxious about my upcoming month.”
• “Even though the simulation was helpful in having me realize how inaccessible resources are in Tanzania, there is no way to adequately prepare someone for the feeling of helplessness I experienced in Tanzania. The simulation prepared me to think more practically and be more resourceful, and also rely on my clinical skills rather than high tech interventions.”
• “Sim was helpful. View on life and death is very different and imbedded into the culture here—resource limitations (i.e., no vents) contribute to some of the feelings of helplessness regarding saving patients.”
• “It is difficult to simulate your experience in Tanzania, but just going through the thought process and frustration was a good exercise to go through prior to going to Tanzania.”
• “Even though I didn't actively see death happening at Tanzania, the simulation prepares me for the pace of response to children that were declining in their state of health.”

The themes that emerged from this survey have been categorized into the following: (1) The simulation was beneficial in managing expectations, and (2) the simulation provided residents with an understanding of the cross-cultural differences in handling pediatric deaths in a resource-limited setting.

## Discussion

We have described the design and evaluation of a simulated patient death case intended to better equip and heighten cross-cultural awareness of pediatric residents going on a global health elective. Pediatric residents based in resource-rich settings may not encounter death often. Postgraduate training programs could consider offering a simulation case like this one to prepare such residents to be conscious of the cultural norms and differences in managing pediatric death in a resource-limited setting. Residents valued how this experience provided them with an appreciation and understanding of a culture with different standards for managing a dying child. Many residents shared how challenging it was to accept the seeming lack of urgency they felt from their Tanzanian counterparts when encountering a dying child. Tanzanian pediatricians and nurses who encounter death more frequently in a resource-limited setting may intentionally perform fewer medical interventions in resuscitating a dying child because of limitations in their hospital's resources and the predictably poor outcomes despite the good intention of wanting to save a child's life. This cultural norm may be misinterpreted as lack of urgency by unfamiliar residents from resource-rich settings. In creating our simulation case, we intentionally incorporated this cultural norm by having the nurse deliberately appear unhurried and vanish from the patient's room for long periods of time as she presumably cared for other sick children in the hospital. A simulated patient death case before departure can help residents from resource-rich settings manage their expectations accordingly and develop cultural awareness of the differences in managing a dying child in resource-limited settings.

Residents valued how the simulation experience helped to better prepare them emotionally for witnessing the frequent deaths of hospitalized children. In the simulation scenario, the case ended as the child developed cardiac arrest, forcing the residents to decide whether to begin or withhold CPR. If CPR was initiated, residents needed to determine an end point for resuscitation in a resource-limited setting where support and equipment might often be lacking. Decision points like this one are frequently encountered during global health electives, and preparation can be key to navigating challenging situations while being sensitive to the local hospital and cultural norms. In all of the simulations we conducted at our institution, our residents started CPR and continued it until the case ended or conversation with the caretaker about end of life occurred. During the debriefing session, the main factor mentioned by residents for why CPR was started was that this was how they had been trained to respond when a child went into cardiac arrest. It is important to let residents know that withholding CPR may at times be more appropriate, which can be counterintuitive for residents trained in resource-rich settings. During the debriefing sessions, these cultural norms were explored to help prepare our residents beforehand for different approaches to managing a dying child, and we encouraged our residents to follow the lead of the local medical providers.

Providing guidance to residents on how to process and deal with child mortality during their elective is important. Within our program, we have a site director at the host institution who met weekly with residents to process and debrief what they had been encountering in the hospital. We also encouraged our residents to debrief with the medical team caring for the dying child if appropriate and to practice reflective journaling. Upon returning to the US, residents met with the program or rotation director for a final debriefing and well-being check-in.

Based on the evaluations, we intend to continue providing this simulated patient death case to prepare residents for their elective. For the critical actions checklist, most residents decide to administer an IV fluid bolus as quickly as possible (i.e., 20 mL/kg over 5 minutes) since the child is in shock. However, when treating a child with severe acute malnutrition, a smaller IV fluid bolus given over a longer period (i.e., 15 mL/kg over 1 hour) is recommended by WHO guidelines to prevent congestive heart failure and pulmonary edema.^15^ Though, based on the data collected, a small proportion of our residents did not find the simulation beneficial, we did not receive any specific feedback as to why. We speculate that perhaps these residents may not have witnessed any child death during their elective. Limitations to the design and evaluation of our module include the lack of a formal needs assessment and the fact that our evaluation was postelective and survey based, with general questions. We did not evaluate whether the residents had retention of knowledge or measure behavior changes after residents had undergone the simulation. Future modifications to the evaluation could include pre- and postsurveys and postelective focus groups, which would allow for measuring transfer of knowledge and specific analysis of any behavior changes. Another limitation was the module's development with high applicability to the setting in Tanzania, which may not be generalizable to other resource-limited settings given that cross-cultural differences can vary. Using a needs assessment with traveling residents and local staff at the host hospital, additional simulated cases could be created that highlight other cultural and management differences in the care of hospitalized children that residents may be unprepared to handle during their elective.

## Appendices


Simulation Case.docxSimulation Images.docxCritical Actions Checklist.docxDebriefing Materials.docxSurvey Instrument.docx
*All appendices are peer reviewed as integral parts of the Original Publication.*
