# Supplementary material for: Child Death in a Resource-Limited Setting: A Simulation Case for Pediatric Residents to Prepare for Global Health Electives
Source: MedEdPORTAL. 2023 Sep 1;19:11341. doi: 10.15766/mep_2374-8265.11341 (PMC10471738; doi:10.15766/mep_2374-8265.11341)
Supplement: Supplementary file 1 — Simulation Case.docxSimulation Images.docxCritical Actions Checklist.docxDebriefing Materials.docxSurvey Instrument.docx [file mep_2374-8265.11341-s001.zip › A. Simulation Case.docx]

| **Appendix A: Simulation Case**  **SIMULATION CASE TITLE: Child Death in a Resource-Limited Setting**  **AUTHORS: Duncan K. Hau MD, Joy D. Howell MD, Adetunbi Ayeni MD, Michael J. Alfonzo MD, Kevin Ching MD**  **LEARNER AUDIENCE: Pediatric Residents** | |
| --- | --- |
| **PATIENT NAME: Dodo**  **PATIENT AGE: 5 years**  **CHIEF COMPLAINT: Diarrhea x 3 days**  **PHYSICAL SETTING: Malnutrition unit at a resource-limited hospital** | |
| **Brief narrative description of case** | A 5-year-old HIV infected child with severe acute malnutrition is brought into the hospital by an orphanage caretaker because of diarrhea x 3 days. He is found to be in compensated hypovolemic shock that rapidly progresses to uncompensated shock and eventually cardiac arrest despite all forms of treatment. |
| **Primary Learning Objectives** | - Reflect on cross-cultural differences that may impact the delivery of healthcare during a global health elective. - Understand different approaches in managing child death in a resource-limited setting. - Manage hypovolemic shock in a child with severe acute malnutrition. |
| **Critical Actions** | - Diagnose severe acute malnutrition - Identify signs and symptoms of shock in a child with severe acute malnutrition - Manage compensated and uncompensated hypovolemic shock in a child with severe acute malnutrition - Recognize limitations of resources and likely outcomes in these settings - Demonstrate teamwork and communication |
| **Learner Preparation or Prework** | - Inform learners the simulation is intended to prepare them for their global health elective - Learners will be working together as a team - Orient the learners to the manikin - Orient the learners that the simulation takes place in the malnutrition unit at a resource-limited hospital |

| **Initial Presentation** | | | |
| --- | --- | --- | --- |
| **Initial vital signs** | The initial vital signs and anthropometric measurements are presented on a clipboard next to the patient’s bed. In the clipboard is also a table for weight-for-height/length from 2 to 5 years for boys to diagnosis severe acute malnutrition.  HR 150, BP 95/50, RR 42, O_2_ 95%, Temp 36C  Weight = 10kg, Height = 100cm | | |
| **Overall Setting and Appearance** | Upon first entering the room there is a sign that reads “Malnutrition Ward”. The child manikin is lying on a bed and with the caretaker sitting next to him. Additional beds with manikins on them are nearby to represent a crowded hospital. | | |
| **Standardized Participants (and their roles in the room at case start**) | The caretaker of the child is present at the beginning. The caretaker is from the orphanage. When asked by the learners, the caretaker will provide information about the child ‘s medical history.  After the learners start talking to the caretaker, the nurse will enter the room. The nurse will ask the learners if they need any medications/supplies and will let the learners know which of these items are available in their setting (see equipment list in Methods). Some items will be empty or low supply (i.e. oxygen tank, glucometer box, adrenaline. The nurse will need to leave the room to gather supplies (supplies should be outside the simulation room). The nurse should deliberately appear unhurried when gathering supplies and be away for long periods to presumably care for other sick children in the hospital.  As the case progresses and the child worsens clinically (tachypneic, hypotensive). The nurse will communicate eventually how the child has become unresponsive to alert the learners the child has gone into cardiac arrest. | | |
| **HPI** | Caretaker will provide the information when asked by the learners.  *Dodo is a 5 year old HIV infected child with diarrhea x 3 days. He has been having up to 10 episodes of non-bloody diarrhea per day. Over the past 24 hours he has been less active and not interested in taking fluids. No fever, vomiting or cough. He has voided once in the past 24 hours. There are other children in the orphanage with diarrhea. He came to the orphanage last week because his mother died. His father died several years ago.*  Learners will obtain further history of past medical history, medications, allergies, and family history.  Learners will then perform a physical examination. On a clipboard next to the bed are the initial vital signs and anthropometric measurement of the child.  Learners may request tests. Test available at the hospital include comprehensive metabolic panel and complete blood count. However, reagents are “out” for the comprehensive metabolic panel, while the complete blood count turnaround time for results will take one day. | | |
| **Past Medical/Surgical History** | **Medications** | **Allergies** | **Family History** |
| HIV | Co-trimoxazole ½ tablet daily | None | Unknown |
| **Physical Examination** | | | |
| **General** | Lethargic and cachectic, too tired to talk or move | | |
| **HEENT** | Sunken eyes, dry mucous membranes | | |
| **Neck** |  | | |
| **Lungs** | Clear to auscultation bilaterally | | |
| **Cardiovascular** | Tachycardiac, normal S1 + S2, regular rhythm, no murmurs  Weak pulses, capillary refills 4-5 seconds | | |
| **Abdomen** | Thin and soft, hyperactive bowel sounds, no tenderness | | |
| **Neurological** |  | | |
| **Skin** | Tenting skin turgor | | |
| **GU** |  | | |
| **Psychiatric** |  | | |

| **Instructor Notes - Changes and CASE Branch Points**  *This section should be a list with detailed description of each step than may happen during the case. If medications are given, what is the response? Do changes occur at certain time points? Should the nurse or other participant prompt the learners at given points? Should new actors or participants enter, and when? Are there specific things the patient will say or do at given times? There are a few examples given, but it is expected that most cases will have many more changes and potential branch points.* | | |
| --- | --- | --- |
| **Intervention / Time point** | **Change in Case** | **Additional Information** |
| Time 0-10 minutes | Learners start with taking history from caretaker and performing physical exam.  Based on history, vital signs and physical exam, learners must recognize patient has severe acute malnutrition and is in compensated hypovolemic shock.  Learners must:   - Request for intravenous line set up and administration of fluids for hypovolemic shock. - Monitor vital signs when fluids start for concern of congestive heart failure in children with severe acute malnutrition. | After the initial history and physical exam, learners may request for diagnostic test, medications, and supplies.   - Diagnostic test available at the hospital include comprehensive metabolic panel and complete blood count. However, reagents are “out” for the comprehensive metabolic panel, while the complete blood count turnaround time for results is one day. - Medications and supplies available are listed in the Methods section.   The nurse should deliberately appear unhurried when gathering supplies and be away for long periods to presumably care for other sick children in the hospital. |
| 10 minutes into the case | Patient develops labored breathing. Respiratory rate increases to 60 breaths per min and oxygen saturation decreases to mid-80s.  Fine crackles are noted on lung exam. Nurse points out jugular venous distention, liver enlargement, galloping heart rhythm and dusky appearance.  Learners should recognize patient has developed congestive heart failure and pulmonary edema from the IV fluids. Learns should stop IV fluids at this point. | Learners will likely inquire about respiratory support available in hospital. Nurse will inform learners of available supplies in the hospital (empty oxygen tank and nasal cannula). |
| 15 minutes into case | Patient has diarrhea noted on bedsheet and becomes hypotensive.  Caretaker will point out the diarrhea in the child’s shorts to learners (after pushing a syringe with brown water).  Nurse will measure blood pressure and report BP 75/39. | Caretaker will push a pre-filled 150ml syringe hidden behind the manikin. Syringe contains brown water (water + brown food color dye) to represent diarrhea and is connected to hidden tubing so that copious diarrhea exits the child’s shorts. |
| 18 minutes into the case | Patient becomes unresponsive and develops cardiac arrest. Nurse will point out the patient is unresponsive and does not have a pulse.  Caretaker in a panicked voice will ask, *“Is Dodo going to die?*” | Learners will need to decide whether to start CPR. They will likely request adrenaline. Nurse will gather supply and let learners know only 2 doses left. |
| Case ends at 20 minutes | Facilitator lets learners know the case ends. |  |

**Ideal Scenario Flow**

The learners enter the “Malnutrition Ward” and perform a history and physical exam. Learners note the patient has severe acute malnutrition based on weight/height, and he is in compensated hypovolemic shock. They request for an intravenous line to be placed and start IV fluids Ringer’s Lactate with 5% glucose 15ml/kg to be given over 1 hour. Patient’s weight is 10kg, so total volume is 150ml. The learners monitor the pulse rate and breathing rate after starting IV hydration and note the patient is deteriorating due the IV fluids causing congestive heart failure and pulmonary edema. The learners will stop the IV fluids. The patient continues to worsen clinically by having persistent diarrhea and develops hypotension. This quickly leads to cardiac arrest. Learners will start CPR, request adrenaline, and begin a conversation with the caretaker that the patient may not survive. The case ends at this point.

**Anticipated Management Mistakes**

1. Intravenous fluids: Learners will generally be unfamiliar with the need to give intravenous fluids over a longer duration for a child with severe acute malnutrition compared to a well-nourished child. Learners will commonly ask for the fluids to be given over a 5-10 minute period, which will lead sooner to congestive heart failure. However, even if the learners request to give the fluids over the appropriate 60 minutes, the patient will inevitably develop congestive heart failure.
2. Oral Rehydration: At the beginning, learners may request to try oral rehydration (ReSoMal) via nasal-gastric tube first instead of intravenous fluids. If learners decide on oral rehydration as initial management, the patient should fail to improve with oral rehydration by continuing to clinically deteriorate.
