# Supplementary material for: Child Death in a Resource-Limited Setting: A Simulation Case for Pediatric Residents to Prepare for Global Health Electives
Source: MedEdPORTAL. 2023 Sep 1;19:11341. doi: 10.15766/mep_2374-8265.11341 (PMC10471738; doi:10.15766/mep_2374-8265.11341)
Supplement: Supplementary file 1 — Simulation Case.docxSimulation Images.docxCritical Actions Checklist.docxDebriefing Materials.docxSurvey Instrument.docx [file mep_2374-8265.11341-s001.zip › B. Simulation Images.docx]

**Appendix B: Simulation Images**


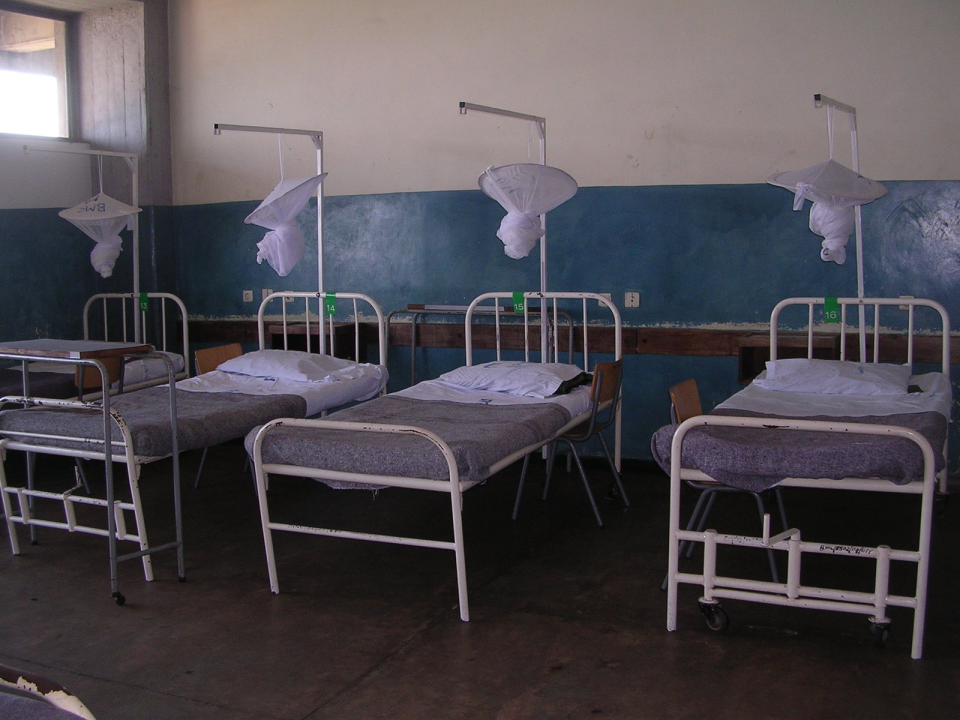

Photo courtesy of Kevin Ching, MD

Figure 1: Malnutrition Ward at Bugando Medical Center in Mwanza, Tanzania. During a busy season or regional drought, beds may be double occupied with children.


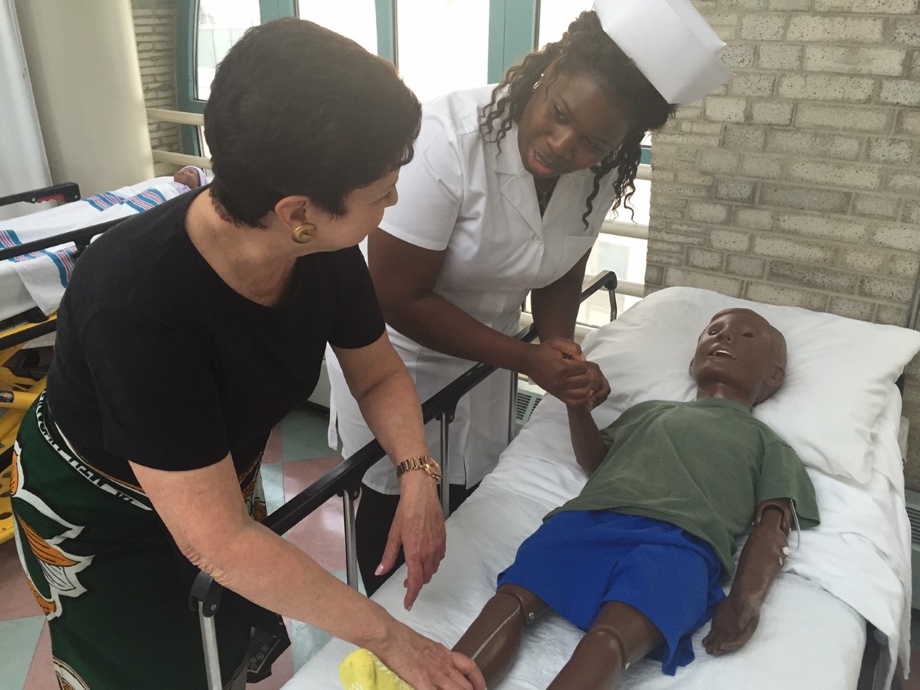

Photo courtesy of Kevin Ching, MD

Figure 2. Simulation picture of pediatric nurse and caretaker discussing about the child. Additional manikins are in the room for crowding.


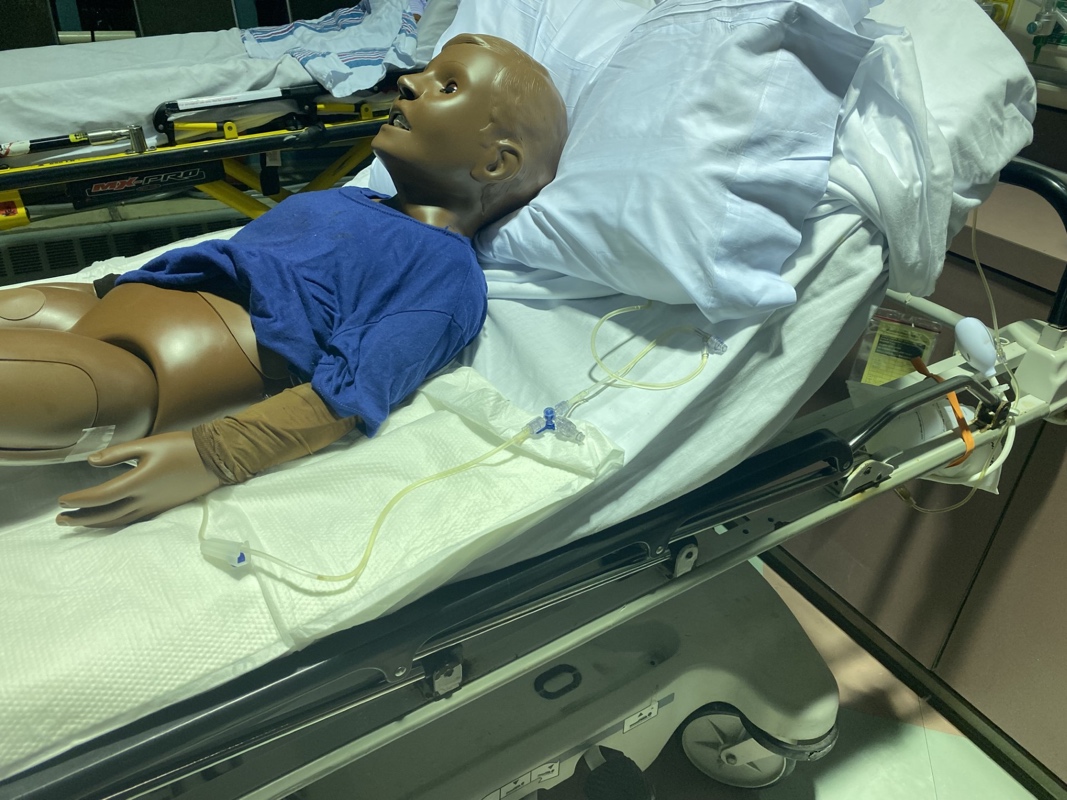

 Photo courtesy of Kevin Ching, MD

Figure 3. Set up for simulated liquid diarrhea. Large volume syringe is behind pillow, which is decompressed by the caretaker during the simulation. Tubing is covered by sheets and manikin’s shorts during the simulation.
