# Supplementary material for: Child Death in a Resource-Limited Setting: A Simulation Case for Pediatric Residents to Prepare for Global Health Electives
Source: MedEdPORTAL. 2023 Sep 1;19:11341. doi: 10.15766/mep_2374-8265.11341 (PMC10471738; doi:10.15766/mep_2374-8265.11341)
Supplement: Supplementary file 1 — Simulation Case.docxSimulation Images.docxCritical Actions Checklist.docxDebriefing Materials.docxSurvey Instrument.docx [file mep_2374-8265.11341-s001.zip › C. Critical Actions Checklist.docx]

**Appendix C: Critical Actions Checklist**

1. **Initial evaluation and management of shock in a child with severe acute malnutrition**
   - Obtains history of present illness, past medical/surgical history, medications, allergies
   - Assesses vital signs
   - Performs physical examination
   - Request for insertion of intravenous line
   - Administer IV fluid at 15ml/kg over 1 hour (Patient’s weight = 10kg -> total fluid 150ml)
   - Pediatricians monitor closely the heart rate and respiratory rate after starting IV fluids
   - Stop IV fluids when patient develops signs of congestive heart failure and pulmonary edema, and request for respiratory support.
2. **Cardiac Arrest**
   - Recognize limitations of resources available in hospital
   - Optional: Begin CPR and request for adrenaline
   - Start conversation of death with caretaker about patient’s condition
3. **Interpersonal Skills**
   - Explains clinical findings and plan to caretaker and involves her in shared decision making
   - Demonstrates professionalism and appropriate communication with pediatric nurse
