# Supplementary material for: Child Death in a Resource-Limited Setting: A Simulation Case for Pediatric Residents to Prepare for Global Health Electives
Source: MedEdPORTAL. 2023 Sep 1;19:11341. doi: 10.15766/mep_2374-8265.11341 (PMC10471738; doi:10.15766/mep_2374-8265.11341)
Supplement: Supplementary file 1 — Simulation Case.docxSimulation Images.docxCritical Actions Checklist.docxDebriefing Materials.docxSurvey Instrument.docx [file mep_2374-8265.11341-s001.zip › D. Debriefing Materials.docx]

**Appendix D: Debriefing Materials**

At the beginning the facilitator should preface this was a case where the patient’s death was expected and inevitable. The facilitator should let learners know that despite appropriate treatments, these patient outcomes are common in resource limited settings.

The facilitator will then let the learners know the debriefing will focus initially on the emotions encountered during the case and then we will address the medical management. The first question to ask should be:

- How did that feel? (Allow each learner a turn to respond. Feelings of failure or frustration are commonly expressed)

The facilitator will ask one learner to summarize the case and ask learners what went well, what would they do differently, decision making at the end of the case, and about the nurse’s demeanor.

- Can someone summarize the case?
- What aspects of the case went well?
- What obstacles did you encounter?
- How did the patient respond differently to your interventions than you expected?
- What was your rationale to start or withhold CPR? If you initiated CPR, what was your endpoint for terminating resuscitation?
- What did you observe about the nurse’s demeanor? How did this make you feel?

The facilitator should highlight how the nurse may have appeared as unhurried during the case. Though this may be interpreted by the residents as lacking urgency in saving a child’s life, nurses and doctors working in resource limited settings are often keenly aware of the few resources available and may intentionally perform fewer medical interventions in resuscitating a dying child because of the limitations. This cultural norm may be misinterpreted by residents from resource-rich settings, but it is important to underscore this difference in approach in order to heighten residents’ cultural awareness and to avoid judgment.

The later period of debriefing will focus on the medical management of shock and severe acute malnutrition. Since learners are unlikely to have taken care of children with severe acute malnutrition in resource-rich settings, the following should be reviewed:

- Diagnosis of severe acute malnutrition

Severe acute malnutrition is defined as severe wasting (Marasmus) diagnosed by weight-for-height/length <-3SD or mid-upper arm circumference < 115 mm), or edema of both feet with or without severe wasting (Kwashiorkor). On examination look for:

- - - Signs of dehydration (i.e., sunken eyes, delayed capillary refill, dry mucous membranes, poor skin turgor)
    - Severe palmar pallor
    - Bilateral pitting edema
    - Signs of vitamin A deficiency (i.e., dry conjunctiva or cornea, Bitot spots, corneal ulceration, keratomalacia)
    - Localizing signs of infection (i.e., ear and throat infections, skin infection or pneumonia)
    - Signs of HIV infection
    - Fever or hypothermia
    - Mouth ulcers
    - Skin changes in Kwashiorkor (i.e., hypo-or hyperpigmentation, desquamation, ulceration, exudative lesions)
- Recognition of dehydration/shock in severe acute malnutrition

Since there are overlapping signs of dehydration/shock and severe acute malnutrition, dehydration/shock tends to be over diagnosed and its severity over estimated in children. It is challenging to determine dehydration accurately from clinical signs alone. Assume children with watery diarrhea or reduced urine output have some degree of dehydration.

Shock consists of first compensated shock (increased heart rate, normotension) followed by uncompensated shock (increased heart rate, hypotension). Hypotension defined in children is:

Age Systolic Pressure (mmHg)

Term Neonates < 60

Infants < 70

Children 1–10 years < 70 + (age in years x 2)

Children > 10 years < 90

- Treatment of shock in a child with severe acute malnutrition

Give IV fluid at 15ml/kg over 1 hour using any of the following solutions:

- - - Ringer’s lactate with 5% glucose
    - Half-strength Darrow’s solution with 5% glucose
    - 0.45% NaCl plus 5% glucose

The above solutions are recommended by WHO guidelines based on availability and the need for glucose. A small IV fluid bolus given over a longer period (i.e., 15 ml/kg over 1 hour) is recommended by WHO guidelines to prevent congestive heart failure and pulmonary edema. Measure the heart rate and respiratory rate at the start of IV infusion and every 5-10 minutes. If there are signs of improvement, another IV infusion at 15ml/kg over 1 hour can be given, and then switch over to oral rehydration with ReSoMal.

If the child worsens during IV rehydration (increased heart rate, increased respiratory rate, crackles, increased jugular venous pressure, galloping heart rate), IV infusion should be stopped because IV fluids can cause congestive heart failure and pulmonary edema in children with severe acute malnutrition.

The facilitators will then let learners know the importance of processing and dealing with child mortality. Examples we provide to learners include:

- Journaling
- Processing and debriefing with the medical team
- Processing and debriefing with the site director at the host institution
- Processing and debriefing with the program director at the home institution

The facilitators will end by asking all learners one take-home lesson learned from the simulation.

**References**

1. Pocket Book of Hospital Care for Children: Guidelines for the Management of Common Childhood Illnesses. 2nd ed. Geneva: World Health Organization; 2013.
