# Supplementary material for: Child Death in a Resource-Limited Setting: A Simulation Case for Pediatric Residents to Prepare for Global Health Electives
Source: MedEdPORTAL. 2023 Sep 1;19:11341. doi: 10.15766/mep_2374-8265.11341 (PMC10471738; doi:10.15766/mep_2374-8265.11341)
Supplement: Supplementary file 1 — Simulation Case.docxSimulation Images.docxCritical Actions Checklist.docxDebriefing Materials.docxSurvey Instrument.docx [file mep_2374-8265.11341-s001.zip › E. Survey Instrument.docx]

**Appendix E: Survey Instrument**

1. During the course of your elective in Tanzania, how many times did you witness or were present during the death of a patient?

0 1-5 6-10 >10

1. Did you need to participate in a resuscitation event while in Tanzania?

Yes No

1. The pre-departure global health simulation was beneficial to my preparation for the mortality encountered in Tanzania.

Strongly disagree Disagree Neutral Agree Strongly Agree

1. The pre-departure global health simulation was beneficial to my preparation for the emotional aspects encountered in Tanzania.

Strongly disagree Disagree Neutral Agree Strongly Agree

1. The pre-departure global health simulation is a useful tool for global health training.

Strongly disagree Disagree Neutral Agree Strongly Agree

1. Please share your thoughts regarding your real or simulated Tanzania experience?
